# Knowledge and intentions to use fertility preservation among urban Chinese cancer patients: A study from Hong Kong

**DOI:** 10.1371/journal.pone.0307715

**Published:** 2024-09-11

**Authors:** Louis S. Chan, Kim L. Cochon, Tin C. Li, Jacqueline P. W. Chung, Jean H. Kim

**Affiliations:** 1 School of Public Health, Faculty of Medicine, The Chinese University of Hong Kong, Shatin, New Territories, Hong Kong SAR; 2 Institute of Clinical Epidemiology, National Institutes of Health, University of the Philippines - Manila, Manila, Philippines; PLOS: Public Library of Science, UNITED KINGDOM OF GREAT BRITAIN AND NORTHERN IRELAND

## Abstract

**Objective:**

To assess the knowledge levels and fertility preservation (FP) intentions of urban Chinese cancer patients.

**Methods:**

A cross-sectional study was conducted on Hong Kong Chinese male and female cancer patients aged 18–54 years (N = 325) who were recruited by a local non-governmental organization for cancer patients between July 2020 to January 2021. Patients completed a self-administered questionnaire on knowledge, perceptions, and intentions to use FP services/seek FP-related information. Multivariable logistic regression was used to explore the correlates of intention to seek additional FP information and intention to undergo FP treatments.

**Results:**

Although cancer patients demonstrated a good knowledge of the available FP treatment options, they were less knowledgeable about the legal restrictions of these procedures. Only one in seven cancer patients first became aware of FP through a health provider and the majority of cancer patients felt they did not have adequate knowledge about FP to make informed FP decisions at the current time. Yet, over one-third of cancer patients would consider FP options even if their cancer or cancer treatment had < 5% chance of causing infertility, and 13.4% of females and 14.6% of males would delay their cancer treatment by ≥ 3 months to undergo FP procedures. However, for both sexes, the main perceived barrier to obtaining FP was its financial cost. Patients with older-aged spouses were less likely to seek FP treatments or seek more information about FP.

**Conclusion:**

There is an unmet need for more FP information and FP services for reproductive-aged cancer patients in East Asian populations. Greater integration of FP services into cancer treatment requires a reduction of cost barriers, greater provision of timely FP information, and improved referral systems.

## Introduction

The annual incidence of cancer is estimated to be over 18 million cases worldwide and at the current time, approximately one in 4 cancer patients is between the ages of 15–49 years [1–3]. Among reproductive-aged cancer patients, one notable adverse effect of cancer treatments (surgery, radiotherapy, gonadotoxic chemotherapy) and the disease itself is the impairment of fertility and the lower likelihood of having biological offspring [4–6]. Currently, many effective fertility preservation (FP) procedures are available for cancer patients such as ovarian/testicular tissue cryopreservation which has been shown to allow cancer patients to have offspring [7–9]. At this time, standardized national guidelines and international FP recommendations have been published for female cancer patients in various countries [10–14]. Many countries such as the United States, have thereby begun to include FP services in conjunction with cancer treatment [9, 15].

In recent years, the past research on cancer patients undergoing FP has mainly examined the patient characteristics and reproductive outcomes of FP procedures [8, 16–19]. The majority of international research on FP has been conducted on female patients and has indicated that FP is a viable option for many women with early-stage cancers. Moreover, candidates for FP have shown comparable oncologic outcomes as those who did not undergo FP [20, 21]. Studies from Western countries have indicated that younger cancer patients and those with private insurance were more likely to undergo FP [22]. Recently, there has been increasing research interest in the FP decision-making process among cancer patients. It has been noted that the provision of high-quality information and counselling lowered the likelihood of emotional distress, conflicting concerns, and later regret [22, 23]. Studies have noted that cancer patients often do not have adequate information for informed decision-making [24, 25] and that there is a lack of integration between oncological care and FP treatment [26, 27]. However, the majority of studies on FP have been conducted in Western countries with comparatively little research in areas such as East Asia. A recent cross-national study of East Asian countries noted that FP for child/adolescent-aged cancer patients was still in the developing stages due to the inadequate expertise in these FP treatments and lack of organized promotion of FP [28]. Studies from mainland China, in particular, have shown that oncologists rarely referred their cancer patients for FP due to the lack of training in this area.

Despite progress in the effectiveness of FP procedures, the attitudes toward FP are a vastly understudied area of health services research outside of Western countries. In many East Asian cultures as in many traditional cultures, having children is considered important for preserving the family lineage, particularly for males [29, 30]. Yet, in many regions of East Asia, the mean age of marriage has risen to the highest in the world. At the current time, the typical age of first marriage is well over 30 in Hong Kong, Singapore, and South Korea [31–33]. Cancer patients who have not yet had the desired number of children as their age advances may wish to undergo FP services.

In Hong Kong, in-vitro fertilization procedures (IVF) in public hospitals are restricted to married, heterosexual couples, but gamete preservation is permitted for cancer patients regardless of their marital status with the partial subsidy in public hospitals or as an out-of-pocket procedure in private clinics [34, 35]. At the current time, however, there are no standardized protocols or procedures for oncologists to refer their patients to FP services in the public or private sector. It is unknown whether there is an unmet need for greater knowledge provision to cancer patients and whether these patients would be interested in undergoing FP before their cancer treatments. This study seeks to assess the knowledge levels and FP perceptions of Chinese cancer patients in Hong Kong, an East Asian city with increasing cancer rates [36]. It also aims to examine the factors associated with the intent to seek FP services in this patient group and the barriers to undergoing FP procedures in Hong Kong. These findings can inform government policy and possible development of public-private partnership programs for the provision of FP services.

## Methods

This cross-sectional study focused on Chinese adult cancer patients residing in Hong Kong. Between July 2020 and December 2020, outreach workers of a major non-governmental organization (NGO) that provides a centralized collection of cancer information for patients in Hong Kong contacted cancer patients ≥18 years from their cancer database who met the Chinese language and residency eligibility criteria after ethics approval was obtained from The Chinese University of Hong Kong—Survey and Behavioural Research Ethics Committee (CUHK—SBREC) (see S1 and S2 Appendices). Rather than use fliers or mass emails which would limit the response of cancer patients, patients were contacted directly by the outreach workers and given a small coupon incentive (approximately US$ 7). The potential respondents were asked to complete an anonymous, self-administered questionnaire written in English and Chinese (see S3 Appendix), and return it to the workers. Of the contacted patients, 325 consented to join the study (73.8%).

The minimum sample size requirement of the survey was determined by computing the sample size requirement to achieve 80% power when performing multiple logistic regression. Consequently, a minimum sample size of 324 cancer patients was calculated based on the following information: 1) odds ratio of the association between sex and seeking more FP information of 2.0; 2) probability of seeking more FP information among males of 30%; 3) significance level set at 5%; 4) coefficient of determination (r^2^) with other covariates of 0.09; 5) and 5) percentage of male cancer patients of 40%. The computation was performed using G*Power Version 3.1.9.6.

Socio-demographic and work-related information was collected from the patients including age, gender, age of spouse (if applicable), educational attainment, household income, occupation, religious affiliation, type of health insurance possessed, number and sex of any biological children, cancer type, cancer stage, and treatments received for cancer.

Patients were assessed concerning their fertility preservation-related knowledge by asking about their awareness of various FP treatments and their knowledge of FP services available in Hong Kong. A score was computed for fertility preservation knowledge by summing up the number of correct responses for each patient (range 0–9 points). Patients were then asked about their main source of information for the fertility preservation treatments, their perception of the adequacy of information from publicly available sources to make an informed decision about FP treatment, factors that would influence their decision to seek or not seek FP treatments, and their perceived barriers to seeking FP treatments. The questionnaire also inquired about the maximum delay in cancer treatment due to undergoing FP procedures that were acceptable to the patient.

The main outcomes of interest were plans to seek FP services and additional FP information. Patients were asked whether they planned to seek additional information about FP services, and if they had plans to seek FP services in Hong Kong.

Descriptive statistics were reported for sociodemographic data, the proportion of knowledge items answered correctly, and the proportion of patients agreeing with individual attitudinal and perception items. Missing observations were not imputed in this study. Univariable analysis was performed using the chi-square test or Fisher’s exact test to compare percentages of factors according to sex. Variables that had a p < 0.250 in the unadjusted analyses were included as covariates for the backward multivariable logistic regression analyses. Statistical significance was set at α = 0.05. Odds ratios, 95% confidence intervals (CI), and p-values were reported for statistically significant factors. STATA IC version 14.0 [37] was used for all analyses.

Informed consent was received before the completion of the survey and ethics approval was obtained from The Chinese University of Hong Kong—Survey and Behavioural Research Ethics Committee (CUHK—SBREC). This study adhered to the Strengthening the Reporting of Observational Studies in Epidemiology (STROBE) guidelines (see S4 Appendix).

## Results

The background characteristics of the study sample are shown in Table 1. The majority of the study sample are females (87.4% female versus 12.6% male). Among the study sample, over half (56%) were aged ≥ 45 years, while only 5.8% were less than 35 years of age. In terms of other sociodemographic characteristics, most were married/cohabiting (58.8%), had post-secondary or tertiary education (51.1%), were employed (61.5%), were non-religious (52.3%), and had some type of health insurance coverage (70.2%). Although there was a wide range of household incomes, only 28.9% of patients reported above the median monthly household income in Hong Kong. Approximately half of the respondents (51.4%) had no biological children. There were no significant differences between sexes in sociodemographic attributes other than occupation, but the type of cancer varied by sex. Among female patients, 70.1% (199) reported having breast cancers, while 80.5% (33) of male patients reported having other solid tumours (p < 0.001).

**Table 1:** Background characteristics of adult Chinese cancer patients in Hong Kong (N = 325).

Characteristics	Total (N = 325)	Males (n = 41)	Females (n = 284)	P-value
	n (%)	n (%)	n (%)	
**Age (y)**				0.959
18–34	19 (5.8)	2 (4.9)	17 (6.0)	
35–44	124 (38.2)	16 (39)	108 (38)	
45–54	182 (56)	23 (56.1)	159 (56)	
**Education**				0.984
Up to Secondary	159 (48.9)	20 (48.8)	139 (48.9)	
Post-Secondary/Tertiary	166 (51.1)	21 (51.2)	145 (51.1)	
**Marital status**				0.509
Married/Co-habitating	191 (58.8)	21 (51.2)	170 (59.9)	
Single	93 (28.6)	13 (31.7)	80 (28.2)	
Divorced/Separated/Widowed	41 (12.6)	7 (17.1)	34 (12)	
**Working status**				< 0.001
Employed	200 (61.5)	29 (70.7)	171 (60.2)	
Housewife	69 (21.2)	0 (0)	69 (24.3)	
Retired	17 (5.2)	4 (9.8)	13 (4.6)	
Unemployed/Student	38 (11.7)	8 (19.5)	30 (10.1)	
**Religion**				0.836
No religion	170 (52.3)	21 (51.2)	149 (52.5)	
Buddhist	41 (12.6)	6 (14.6)	35 (12.3)	
Catholic	23 (7.1)	4 (9.8)	19 (6.7)	
Protestant	91 (28)	10 (24.4)	81 (28.5)	
**Number of biological children**				0.189
0	167 (51.4)	17 (41.5)	150 (52.8)	
1	81 (24.9)	10 (24.4)	71 (25)	
≥ 2	69 (21.2)	13 (31.7)	56 (19.7)	
No information	8 (2.5)	1 (2.4)	7 (2.5)	
**Monthly household income (USD)**				0.846
< 2,548	87 (26.8)	12 (29.3)	75 (26.4)	
2,548–5,095	142 (43.7)	16 (39)	126 (44.4)	
≥ 5,096	94 (28.9)	12 (29.3)	82 (28.9)	
No information	2 (0.6)	1 (2.4)	1 (0.4)	
**Type of health insurance**				0.030
None	97 (29.8)	18 (43.9)	79 (27.8)	
Public	90 (27.7)	13 (31.7)	77 (27.1)	
Private/Both	138 (42.5)	10 (24.4)	128 (45.1)	
**Type of cancer**				< 0.001
Breast	199 (61.2)	0 (0)	199 (70.1)	
Other solid tumours	83 (25.5)	33 (80.5)	50 (17.6)	
Reproductive	25 (7.7)	0 (0)	25 (8.8)	
Blood cancers	18 (5.5)	8 (19.5)	10 (3.5)	
**Cancer stage at diagnosis**				< 0.001
0–1	96 (29.5)	5 (12.2)	91 (32)	
2	110 (33.9)	8 (19.5)	102 (35.9)	
3	50 (15.4)	8 (19.5)	42 (14.8)	
4	50 (15.4)	15 (36.6)	35 (12.3)	
No information/unsure	19 (5.9)	5 (12.2)	14 (4.9)	
**Treatments received**				
Cancer Surgery	71 (21.8)	9 (22)	62 (21.8)	0.986
Radiotherapy	38 (11.7)	3 (7.3)	35 (12.3)	0.351
Chemotherapy	46 (14.2)	7 (17.1)	39 (13.7)	0.566
Others	52 (16)	4 (9.8)	48 (16.9)	0.243
**Spouse’s Age (y)**				< 0.001
18–34	10 (3.1)	5 (12.2)	5 (1.8)	
35–44	51 (15.7)	10 (24.4)	41 (14.4)	
45–54	97 (29.9)	5 (12.2)	92 (32.4)	
≥ 55	30 (9.2)	0 (0)	30 (10.6)	
Not applicable	137 (42.2)	21 (51.2)	116 (40.9)	
**Friends/relatives had FP?**	51 (15.8)	3 (7.3)	48 (17)	0.111

FP-related knowledge levels and information sources are shown in Table 2. Most of the patients had heard about oocyte and sperm cryopreservation, with females having significantly higher awareness of oocyte freezing compared to males (p < 0.05). However, respondents were less aware of other FP options such as embryo cryopreservation. In particular, only 5.2% of patients were aware of radiation shielding. Although two-thirds of patients were aware that private fertility preservation clinics could treat married patients of any age, the vast majority of patients were unaware that public hospitals had stringent age/clinical eligibility criteria for patients seeking FP/assisted reproductive technology services. Furthermore, about half of the patients incorrectly believed that paid gestational surrogacy was available in Hong Kong.

**Table 2:** Knowledge of FP among adult Chinese cancer patients in Hong Kong (N = 325).

Knowledge items	Males (n = 41)	Females (n = 284)	P-value
	n (%)	n (%)	
**Have you heard of the following FP treatment:**			
Oocyte freezing	31 (75.6)	253 (89.1)	0.015
Sperm freezing	30 (73.2)	242 (85.2)	0.051
Embryo freezing	24 (58.5)	167 (58.8)	0.974
Ovarian/testicular tissue freezing	12 (29.3)	56 (19.7)	0.160
Fertility-sparing surgeries	5 (12.2)	66 (23.2)	0.110
Radiation shielding	0 (0)	17 (6)	0.144
**Correctly answered questions about FP services availability**			
Any adult can access fertility preservation treatments in public hospitals without restrictions (False)	4 (9.8)	30 (10.6)	0.875
Any married adult can have FP in private clinics and private hospitals in HKG (True)	31 (75.6)	186 (65.5)	0.199
Paid gestational surrogacy is permitted in HKG (False)	15 (36.6)	134 (47.2)	0.203
**Mean total knowledge score ± SD (maximum = 9 points)**	3.7 ± 1.9	4.1 ± 1.6	0.213
**First source of FP information:**			0.024
Media/news/newspapers/magazines	16 (39.0)	132 (46.5)	
Internet searches	12 (29.3)	36 (12.7)	
Doctors/nurses	6 (14.6)	40 (14.1)	
Friends/Relatives	2 (4.9)	50 (17.6)	
Others	3 (7.3)	14 (4.9)	
Missing	2 (4.9)	12 (4.2)	
**Currently feel able to make well-informed FP decisions**	7 (17.1)	66 (23.2)	0.371
**Most useful format for FP information**			
Talks/lectures/symposiums by specialists	5 (12.2)	53 (18.7)	0.312
Informational websites	5 (12.2)	32 (11.3)	0.796
Videos	5 (12.2)	31 (10.9)	0.791
Printed informational pamphlets	2 (4.9)	35 (12.3)	0.197
Others	0 (0)	8 (2.8)	0.602

When queried as to the first source of their FP information, mass media sources were the most commonly reported source of information. Obtaining information from friends/relatives was also commonly reported. However, information from medical professionals was reported only about one in seven patients. Only a minority of respondents (17.1% of males and 23.2% of females), however, felt that they currently possessed sufficient information to make well-informed decisions about FP treatments. In terms of the most useful format for delivering FP information, 18.7% of females reported that live talks/presentations/symposiums were the most useful, while 12.2% of males reported that live talks, informational websites, and videos were the most useful. The other sources of FP information reported were institutions like the Hong Kong Breast Cancer Foundation and participation in studies related to FP.

The perceptions of FP in this cancer patient study sample are shown in Table 3. The results indicate that even if a disease had a 5% chance of causing infertility, over one-third of men and women would consider undergoing FP services; moreover, an even higher proportion would want their doctor to discuss FP options if their cancer treatment had a 5% risk of causing infertility. These percentages rose with a higher probability of infertility from the disease or treatment. When asked about the factors that would influence their decision to undergo FP treatments, age, financial considerations/cost, and cancer-related factors (prognosis, time available before starting cancer therapy, type of cancer) were the most cited factors followed by their desire to have children and marital status. Religious beliefs as being influencing factors were only reported by 22% of men and 28.5% of women.

**Table 3:** FP-related attitudes among adult Chinese cancer patients in Hong Kong (N = 325).

Factors	Males (n = 41)	Females (n = 284)	P-value
	n (%)	n (%)	
**Would consider FP if chance of infertility due to cancer is:**			
> 1/3 chance of causing infertility	21 (51.2)	160 (56.3)	0.537
About 20% chance of causing infertility	16 (39)	137 (48.2)	0.269
≤ 5% chance of causing infertility	14 (34.2)	104 (36.6)	0.758
**Would consider FP if chance of infertility due to treatment is:**			
>1/3 chance of causing infertility	21 (51.2)	209 (73.6)	0.003
About 20% chance of causing infertility	17 (41.5)	191 (67.3)	0.001
< 5% chance of causing infertility	16 (39)	158 (55.6)	0.046
**Factors that influence decision to have FP**			
Your age	32 (78.1)	257 (90.5)	0.018
Time available before cancer treatment	31 (75.6)	245 (86.3)	0.075
Type of cancer	26 (63.4)	231 (81.3)	0.008
Prognosis of the cancer condition	30 (73.2)	250 (88)	0.010
Financial resources at hand	36 (87.8)	252 (88.7)	0.861
Strong desire to have children	31 (75.6)	233 (82.0)	0.324
Marital status	26 (63.4)	216 (76.1)	0.083
Cost	34 (82.9)	247 (87)	0.479
Religion	9 (22)	81 (28.5)	0.380
Others	5 (12.2)	39 (13.7)	0.788
**Most important factor that influences decision to seek FP**			0.563
Strong desire to have children	14 (34.2)	85 (29.9)	
Your age	5 (12.2)	64 (22.5)	
Cost	7 (17.1)	34 (12)	
Type of cancer	5 (12.2)	44 (15.5)	
Financial resources at hand	4 (9.8)	18 (6.3)	
Marital status	2 (4.9)	13 (4.6)	
Time available before cancer treatment	2 (4.9)	10 (3.5)	
Religion	1 (2.4)	2 (0.7)	
Physical condition	0 (0)	2 (0.7)	
No information	1 (2.4)	12 (4.2)	
**Factors that hinder seeking FP**			
Lack of money/financial support	31 (75.6)	221 (77.8)	0.752
FP hormone/drugs might worsen cancer	21 (51.2)	212 (74.7)	0.002
Would be too weak from cancer/treatments to raise children	22 (53.7)	184 (64.8)	0.167
Delays in starting cancer treatment	20 (48.8)	195 (68.7)	0.012
Desire to avoid additional FP-related surgery	19 (46.3)	160 (56.3)	0.229
**Maximum delay in treatment to consider undergoing FP services**			0.167
Less than 2 weeks	11 (26.8)	115 (40.5)	
2–3 weeks	9 (22)	67 (23.6)	
1–2 months	15 (36.6)	62 (21.8)	
3 months or more	6 (14.6)	38 (13.4)	
No information	0 (0)	2 (0.7)	

When asked what would be the most important factor for deciding on whether to undergo FP services, the desire to have children was ranked first followed by their age. Lack of financial resources was cited as the most important reason that respondents would not seek FP services. Additionally, 63.4% of the patients were unwilling to conceive a child while undergoing cancer treatment. Other reasons cited by more than half of the respondents were concerns about FP hormonal/procedures worsening cancer prognosis, desire to avoid additional surgery from FP treatment, and concerns about delaying cancer treatment. About two-thirds of the women and approximately half of the men would delay their cancer treatment by no more than 3 weeks to receive FP services, but only 13.4% of females and 14.6% of males would delay their cancer treatment by ≥ 3 months to receive FP services.

Of the study sample, 28.6% of males and 17.3% of female patients reported planning to seek more FP information. Although various sociodemographic, perceptual, and clinical factors were associated with the intention to seek more FP information in the unadjusted analysis, only higher education, practicing Buddhism, the younger age of the spouse, and having received cancer surgery or chemotherapy treatment were independently associated with the intention to seek additional FP information in the multivariable analysis (see Table 4). Cancer type and cancer stage were not associated with the intention to seek more information.

**Table 4:** Socio-demographic and clinical correlates of intention to seek more FP information among adult Chinese cancer patients in Hong Kong (N = 296).

Factors	Intention to seek more FP information	Unadjusted model		Adjusted model ^a^	
	n (%)	OR (95% CI)	P-value	OR (95% CI)	P-value
**Sex**					
Male	10 (28.6)	Ref			
Female	46 (17.6)	0.5 (0.2–1.2)	0.125		
**Age (y)**					
18–34	7 (38.9)	Ref			
35–44	30 (27.3)	0.6 (0.2–1.7)	0.317		
45–54	19 (11.3)	0.2 (0.1–0.6)	0.003		
**Education**					
Up to Secondary	16 (11.2)	Ref		Ref	
Post-Secondary/Tertiary	40 (26.1)	2.8 (1.5–5.3)	0.001	3.7 (1.7, 8.1)	0.001
**Marital status**					
Married/Co-habitating	31 (17.7)	Ref			
Single	21 (25)	1.5 (0.8–2.9)	0.172		
Divorced/Separated/Widowed	4 (10.8)	0.6 (0.2–1.7)	0.310		
**Employment**					
Employed	33 (17.9)	Ref			
Unemployed (includes housewife, student, etc.)	23 (20.5)	1.2 (0.7–2.1)	0.580		
**Religion**					
No religion	28 (18.4)	Ref		Ref	
Buddhist	13 (36.1)	2.5 (1.1–5.5)	0.024	3.5 (1.4, 8.8)	0.008
Catholic	5 (22.7)	1.3 (0.4–3.8)	0.631	0.8 (0.2, 2.7)	0.680
Protestant	10 (11.6)	0.6 (0.3–1.3)	0.173	0.5 (0.2, 1.1)	0.079
**Number of biological children**					
0	34 (21.9)	Ref			
1	12 (15.8)	0.7 (0.3–1.4)	0.274		
≥ 2	10 (15.4)	0.6 (0.3–1.4)	0.270		
**Monthly household income (USD)**					
< 2,548	15 (18.8)	Ref			
2,548–5,095	22 (17.2)	0.9 (0.4–1.9)	0.774		
≥ 5,096	19 (21.6)	1.2 (0.6–2.5)	0.647		
**Type of health insurance**					
None	24 (27.6)	Ref			
Public	13 (15.9)	0.5 (0.2–1.1)	0.068		
Private/Both	19 (15)	0.5 (0.2–0.9)	0.025		
**Chance of infertility due to disease**					
> 33%	39 (23.6)	2.1 (1.1–3.9)	0.022		
20%	34 (23.9)	1.9 (1.0–3.4)	0.036		
< 5%	28 (25.7)	2.0 (1.1–3.5)	0.025		
**Chance of infertility due to treatment**					
> 33%	46 (21.8)	2.1 (1.0–4.4)	0.050		
20%	42 (21.9)	1.8 (0.9–3.5)	0.080		
< 5%	35 (22)	1.6 (0.9–2.8)	0.145		
**Type of Cancer**					
Reproductive/Prostate	5 (20.8)	Ref			
Other solid tumours	50 (19)	0.9 (0.3–2.5)	0.828		
Blood cancers	1 (11.1)	0.5 (0.0–4.7)	0.526		
**Cancer Stage**					
0–1	17 (18.7)	Ref			
2	22 (20.6)	1.1 (0.6–2.3)	0.740		
3	9 (18.4)	1.0 (0.4–2.4)	0.964		
4	8 (16.3)	0.8 (0.3–2.1)	0.729		
**Treatments received**					
Cancer surgery	25 (43.1)	5.1 (2.7–9.6)	< 0.001	3.4 (1.1–11.0)	0.037
Radiotherapy	12 (37.5)	3.0 (1.4–6.6)	0.006	0.8 (0.2–2.6)	0.675
Chemotherapy	19 (46.3)	5.1 (2.5–10.3)	< 0.001	3.5 (0.9–13.4)	0.071
Others	5 (11.6)	0.5 (0.2–1.4)	0.193	0.2 (0.1–0.7)	0.008
**Spouse’s age (years)**					
< 45	17 (31.5)	Ref			
≥ 45	14 (11.9)	0.3 (0.1–0.7)	0.003	0.3 (0.1–0.7)	0.010
No partner/spouse	25 (20.2)	0.5 (0.3, 1.1)	0.105	0.6 (0.2, 1.3)	0.164

OR, odds ratio; CI, confidence interval; Ref, reference; USD, United States dollar

^a^ Results presented for factors included in the final logistic regression model only

Of cancer patients. 14.3% of males and 6.9% of females reported their current intention to seek FP services (see Table 5). Intention to seek FP services was not statistically significantly associated with sociodemographic factors such as income, education, or insurance status or with the number of biological children, type of cancer, or stage of cancer. Those who felt that their chance of infertility was less than 5% from their disease were significantly more likely to have the intention to seek FP services (adjusted OR = 6.6, p = 0.001). Additionally, those without partners or with spouses over the age of 45 years were much less likely to seek FP services (adjusted OR = 0.2, p < 0.05).

**Table 5:** Socio-demographic and clinical correlates of intention to seek FP services among adult Chinese cancer patients in Hong Kong (N = 296).

Factors	Intention to have FP services	Unadjusted model		Adjusted model ^a^	
	n (%)	OR (95% CI)	P-value	OR (95% CI)	P-value
**Sex**					
Male	5 (14.3)	Ref			
Female	18 (6.9)	0.4 (0.2–1.3)	0.134		
**Age (y)**					
18–34	1 (5.6)	Ref			
35–44	13 (11.8)	2.3 (0.3–18.6)	0.442		
45–54	9 (5.4)	1.0 (0.1–8.1)	0.972		
**Education**					
Up to Secondary	10 (7)				
Post-Secondary/Tertiary	13 (8.5)	1.2 (0.5–2.9)	0.630		
**Marital status**					
Married/Co-habitating	17 (9.7)	Ref			
Single	3 (3.6)	0.3 (0.1–1.2)	0.096		
Divorced/Separated/Widowed	3 (8.1)	0.8 (0.2–3.0)	0.762		
**Employment**					
Employed	14 (7.6)	Ref			
Unemployed (includes housewife, student, etc.)	9 (8)	1.1 (0.4–2.5)	0.894		
**Religion**					
No religion	9 (5.9)	Ref			
Buddhist	3 (8.3)	1.4 (0.4–5.6)	0.596		
Catholic	2 (9.1)	1.6 (0.3–7.9)	0.571		
Protestant	9 (10.5)	1.9 (0.7–4.9)	0.208		
**Number of biological children**					
0	9 (5.8)	Ref			
1	8 (10.5)	1.9 (0.7–5.2)	0.203		
≥ 2	6 (9.2)	1.6 (0.6–4.8)	0.362		
**Monthly household income (USD)**					
< 2,548	5 (6.3)	Ref			
2,548–5,095	10 (7.8)	1.3 (0.4–3.9)	0.672		
≥ 5,096	8 (9.1)	1.5 (0.5–4.8)	0.494		
**Type of health insurance**					
None	9 (10.3)	Ref			
Public	6 (7.3)	0.7 (0.2–2.0)	0.491		
Private/Both	8 (6.3)	0.6 (0.2–1.6)	0.287		
**If chance of infertility due to disease is:**					
> 33%	13 (7.9)	1.0 (0.4–2.4)	0.938	0.6 (0.2–2.5)	0.516
20%	12 (8.5)	1.2 (0.5–2.8)	0.675	0.6 (0.2–2.8)	0.555
< 5%	15 (13.8)	3.6 (1.5–8.7)	0.005	6.6 (2.1–20.9)	0.001
**If chance of infertility due to cancer treatment is:**					
> 33%	15 (7.1)	0.7 (0.3–1.8)	0.504		
20%	13 (6.8)	0.7 (0.3–1.6)	0.385		
< 5%	15 (9.4)	1.7 (0.7–4.1)	0.254		
**Type of Cancer**					
Reproductive	3 (12.5)	Ref			
Other solid tumours	19 (7.2)	0.5 (0.1–2.0)	0.359		
Blood cancers	1 (11.1)	0.9 (0.1–9.7)	0.913		
**Cancer Stage**					
0–1	7 (7.7)	Ref			
2	11 (10.3)	1.4 (0.5–3.7)	0.529		
3	2 (4.1)	0.5 (0.1–2.6)	0.414		
4	3 (6.1)	0.8 (0.2–3.2)	0.731		
**Treatments received**					
Cancer surgery	12 (20.7)	5.4 (2.2–12.9)	< 0.001	8.4 (1.9–36.4)	0.005
Radiotherapy	6 (18.8)	3.4 (1.2–9.2)	0.019	1.5 (0.3–6.8)	0.609
Chemotherapy	7 (17.1)	3.1 (1.2–8.0)	0.022	0.6 (0.1–2.9)	0.522
Others	3 (7)	0.9 (0.2–3.1)	0.834	0.5 (0.1–2.0)	0.298
**Spouse’s age (years)**					
< 45	11 (20.4)	Ref		Ref	
≥ 45	6 (5.1)	0.2 (0.1–0.6)	0.004	0.2 (0.0–0.6)	0.004
No partner/spouse	6 (4.8)	0.2 (0.1–0.6)	0.003	0.2 (0.1–0.5)	0.003

OR, odds ratio; CI, confidence interval; Ref, reference; USD, United States dollar

^a^ Results presented for factors included in the final logistic regression model only

## Discussion

This study noted that although only about one in thirteen of the cancer patients reported current intention to seek FP services, one-third of our sample would seriously consider FP procedures if their cancer or cancer treatments had even a 5% risk of impairing fertility. Moreover, over one-half of male patients and one-third of female patients would consider delaying their cancer treatment by one month or more to undergo FP procedures. These findings indicate the high value that Hong Kong Chinese cancer patients place upon the ability to have biological offspring. Our results corroborate the cultural acceptance of FP in Hong Kong and that there is a strong interest in FP services and counselling [38]. As in other regions of China, the study findings suggest a large unmet demand for these oncofertility services [39–42] and a large number of barriers to seeking FP services.

An international review noted that lack of information provision, concerns about delaying cancer treatment, and/or aggravation of cancer with hormonal FP treatments were important deterrents to seeking FP [43]. Similar to findings from abroad, our study respondents cited the lack of access to timely information on their fertility preservation options, possible risks in delaying cancer treatment, and the concerns about the adverse effects of FP on their cancer prognosis as major barriers to FP treatment [15]. Hence, our results indicate that Hong Kong cancer patients would benefit from the early provision of evidence-based FP information to make an informed evaluation of the risks and benefits of pursuing FP. However, at the current time, there are no comprehensive information sources for cancer patients who may be interested in FP in Hong Kong. Although Hong Kong cancer patients in this study were generally knowledgeable about the various types of FP procedures, they were much less aware of the restricted availability of FP services in Hong Kong, particularly in the public sector. For instance, Hong Kong cancer patients who do not fulfil various criteria (e.g., age < 35 years, childless, cancer survival rate > 50%, no previous pelvic radiotherapy) must seek FP services in the private sector; at the current time [44]. Clinical counselling should therefore include information not only about the risks and benefits of FP treatment but also inform patients about the eligibility criteria for FP services in the public and private sectors. Since the majority of our study respondents felt they had insufficient information about FP, cancer patients in Hong Kong require medical consultation services and easily accessible information. FP counselling has been also shown to reduce psychological stress and improve the quality of life for cancer patients [45, 46]. Studies from abroad have also investigated the usefulness of various methods of FP education, including informational websites and online decision aids [47, 48]. The provision of FP counselling with supplementary decision aids has been shown to increase knowledge, improve FP decision-making, and reduce future regret [23, 48–51]. Development of Chinese language websites with culturally relevant decision aids should therefore be considered as a cost-effective, solution for the provision of free, reliable FP information in Hong Kong.

In addition to increasing access to FP information sources, there have been international calls for greater integration of FP into oncology treatments, particularly for cancers of the reproductive system [52–55]. Identifying and treating cancer patients who are suitable for FP services requires integration of care between oncologists and FP specialists. The addition of FP services to cancer treatment for women in the UK has been shown to have high satisfaction levels [56]. Yet, even in countries such as the US and Canada, fertility centres were noted to have low levels of oncology referrals [55]. In China, there was also a very low uptake of FP services among cancer patients which was largely attributed to the absence of integrated referral pathways. Studies from abroad also noted that factors such as inadequate staff training and lack of FP staff in oncology centres were impediments to the provision of FP services [15]. Furthermore, oncology physicians were noted to possess limited knowledge of FP treatments and seldom provided written FP information to their cancer patients, and countries such as China rarely even discussed FP options, mainly due to a lack of expertise and absence of training in FP [57–60]. In Hong Kong, knowledge of elective reproductive health services is notably poor, thus requiring additional consultation [61]. Moreover, Chinese cancer patients were noted to desire other forms of information in addition to FP such as advice on improving sexual functioning after cancer treatment, indicating the need for greater integration of clinical services [62].

Although this study did not examine the knowledge, attitudes, and practices of healthcare professionals, past studies from other parts of the world have noted low levels of referral for FP services and healthcare professionals’ lack of detailed knowledge of FP [60, 63]. Our results appear to corroborate findings from other parts of the world. It was shown that only a small proportion of Hong Kong cancer patients first obtained FP information from healthcare staff. At the current time, in Hong Kong, there are no formal linkage or referral protocols between oncology and FP services. Referrals are given at the discretion of the oncologist or if requested by the patients. Healthcare professionals in both oncology and fertility services may thereby benefit from the implementation of training programs and clinical protocols that improve the integration between these specialties. It may be necessary to include these topics in continuing medical and nursing education in Hong Kong to promote collaborative healthcare decision-making for cancer patients.

The last major barrier to greater uptake of FP services by cancer patients in our study was the financial costs of FP. Financial costs were cited as the biggest deterrent in seeking FP services by both sexes in our Hong Kong sample. In the private sector, the cost of oocyte cryopreservation (including drugs, egg retrieval, and freezing) is approximately US$ 12,820 with annual storage costs of another US$ 1500. While the public sector offers services for much lower costs, there is usually a longer waiting time for services (leading to time delays in cancer treatment) and restricted access to some services to married couples under age 40. Hence. public-private-partnership (PPP) programs may be a strategic method of improving manpower efficiency, reducing waiting times, and reducing costs while increasing accessibility of FP services in Hong Kong to more cancer patients. Countries such as China, which may face an increasing demand for oncofertility services in the future may also consider PPP options for the provisioning of FP services to cancer patients.

This study has several limitations. First of all, the small number of male cancer patients included in this study may not be fully representative of the perceptions of Chinese male cancer patients in the region. However, our findings largely corroborated those from international studies, and the biases are likely to be moderate. The study was also conducted based on the recruitment of workers in a single cancer NGO which may introduce some selection bias. However, this cancer NGO is a major centre for cancer patient information in Hong Kong and attracts a wide spectrum of patients seeking information. Nonetheless, we cannot preclude the possibility that there could be selection bias for more educated patients or those with more health information-seeking behaviours. Yet, the response rates of the contacted individuals were relatively high; moreover, our findings largely corroborated those from international studies and mirror findings from mainland China, suggesting that any such biases are moderate. The instrument used in this study has not been validated instrument in Hong Kong but this should not present significant recall bias since the majority of the questions inquired about factual information such as demographic data, aspects of the patient’s cancer and cancer treatment, knowledge of various FP treatments and future intentions to seek FP. Also, given the anonymous nature of the data collection, biases from social desirability should be low. Additionally, the cross-sectional study only examined behavioural intentions and did not follow up to determine whether the patients eventually underwent FP treatments. Future studies should follow patients to determine the factors that predict FP service use. Nonetheless, this study highlights some key health services issues for cancer patients such as the provision of FP information, and the lack of standardized protocols for FP referral. Our results strongly suggest that there is an unmet need for FP referrals and FP counselling as part of standard care for urban Chinese cancer patients. Future studies should examine the knowledge and perceptions of healthcare workers in the region toward oncofertility treatments. Finally, a deeper examination of the culture-specific factors that influence Chinese cancer patient’s FP decision-making warrants further research.

## Supporting information

S1 Appendix CUHK—SBREC initial research ethics approval. (PDF)

S2 Appendix CUHK—SBREC final research ethics approval. (PDF)

S3 Appendix Fertility preservation questionnaire. (PDF)

S4 Appendix STROBE checklist. (PDF)

S1 File Minimal study dataset. (CSV)
