# Supplementary material for: Knowledge and intentions to use fertility preservation among urban Chinese cancer patients: A study from Hong Kong
Source: PLoS One. 2024 Sep 11;19(9):e0307715. doi: 10.1371/journal.pone.0307715 (PMC11389933; doi:10.1371/journal.pone.0307715)
Supplement: S1 Appendix — (PDF) [file pone.0307715.s001.pdf]

THE CHINESE UNIVERSITY OF HONG KONG

M E M O

To : Prof. KIM, Jean Hee
The Jockey Club School of Public Health and Primary Care

From : Secretary
Survey and Behavioural Research Ethics Committee (SBREC)

Tel. : 3943 4209

Date : 9 July 2020

Survey and Behavioural Research Ethics
Reference No. SBRE-19-776

I write to inform you that the Survey and Behavioural Research Ethics Committee has granted approval in principle for you to conduct the surveys or observation of human behaviour by non-clinical means as declared in the application for the following research:

Project Title : Attitude towards fertility preservation in cancer survivors and their partners

Source of Funding : Nil

Reference, if any : Nil

Kindly be reminded that you should also obtain approval from other research ethics committees within the University (e.g., Clinical Research Ethics Committee, Animal Experimentation Ethics Committee) if any parts of your research do not fall under the scope of our Committee. Thank you for your attention.

Alice Hung

c.c. Panel Secretary concerned
