# Supplementary material for: Knowledge and intentions to use fertility preservation among urban Chinese cancer patients: A study from Hong Kong
Source: PLoS One. 2024 Sep 11;19(9):e0307715. doi: 10.1371/journal.pone.0307715 (PMC11389933; doi:10.1371/journal.pone.0307715)
Supplement: S3 Appendix — (PDF) [file pone.0307715.s003.pdf]

Fertility Preservation Questionnaire

The ovaries and testes are the vital organs in the human reproductive system which is most sensitive to injury from medications, diseases, chemotherapy and radiation. Individuals can be exposed to these agents from treatment of cancers and other medical conditions such as autoimmune disease like systemic lupus erythematosus and haematological diseases, with subsequent gonadal damage and infertility. Those who survived the treatment and are planning to raise or complete their family are faced with the problems of gonadal injury and possible infertility in the background of advancing age. If their fertility can be preserved before such treatment is performed then they would have the option to choose to regain their fertility after treatment. If damage to reproductive organs from treatment is likely, freezing gametes, embryos or gonadal tissue may help to preserve fertility or hormonal function. Fertility preservation refers to strategies aimed at preserving one's fertility as well as hormonal function against the damage of gonadotoxic treatment that could render the patient infertile.

We are now conducting a survey to assess the patient's awareness, behavior and knowledge on fertility preservation. The information will be of great value in setting up a fertility preservation service in Hong Kong in the near future. We hope that you can generously spare 5-10 minutes to complete the questionnaire. If you decide to participate in this study after reading and fully understood the relevant information given to you, please complete the questionnaire and mail it back to us in the attached self-addressed stamped envelope. Your participation is totally voluntary. All data collected are for the sole purpose of this research and is treated as highly confidential. You have the right to withdraw from the study at any time. If you have any queries concerning this survey or on fertility preservation, please feel free to contact the undersigned. Thank you very much!

Yours sincerely,

Name and contact of investigator

「保存生殖能力」的問卷調查

人類的卵巢和睾丸是重要的生殖器官,但藥物、疾病、化療或電療都有可能導致其功能大大受損。例如曾經接受癌症治療的患者,或患有自身免疫性疾病如紅斑狼瘡症和血癌病人的生殖能力都有可能下降,甚至不育。隨着醫療技術水平不斷提升,更精準的癌症診斷與疾病治療都令年輕患者存活率增加,當中更有不少打算生兒育女,組織家庭。如果能在進行影響生殖能力的治療前替他們凍存配子、胚胎、卵巢或睾丸組織,便能以此技術去保存他們的生殖能力及增加將來生兒育女的機會。

「保存生殖能力」是透過醫療程序為因接受抗癌療程或疾病而引至生殖能力受損的患者保留內分泌功能及保存其生殖能力。抗癌療程如化療或電療對卵巢和睪丸的功能會造成一定的影響,其損害程度取決於患者的年齡、治療藥物的劑量和療程的種類。

我們現在正進行一項調查,以評估大眾對生育保存的意識,知識和行為。這些信息會為在不久的將來設立在香港保存生殖能力的服務將有很大的價值。我們希望您能騰出 5-10 分鐘來完

成這份問卷調查。若閣下現在充份了解有關是項研究的資料而又決定參與,請閣下完成問卷後,並放進附上的回郵信封寄給我們。所有與此項研究有關的資料,均會保密處理。您的參與全是自願性質。如果您對本次調查或保存生育能力的治療有任何疑問,請隨時與我聯繫!非常感謝你的參與。

此致,
調查員姓名和聯繫方式

Part I: Sociodemographic Data (Please check the appropriate box)
第一部分: 社會人口統計數據 (請選擇合適的答案)

**1) Sex 性別**	☐ Male 男 ☐ Female 女
**2) Marital status 婚姻狀況**	☐ Single 單身<br>☐ Divorced/separated 離婚/ 分居<br>☐ Married/cohabiting 已婚/ 同居<br>☐ Widowed 喪偶
**3) Education 教育程度**	☐ Primary level or less 小學或以下<br>☐ Secondary level 中學 ☐ Postsecondary 預科<br>☐ Tertiary level or more 大學或以上
**4) Occupation 職業**	☐ Full-time job 全職工作 ☐ Student 學生<br>☐ Part-time job 兼職工作 ☐ Retired 退休<br>☐ Housewife 家庭主婦 ☐Unemployed 待業<br>☐ Others 其他(please specify 請註明): ____________________
**5) Religion 宗教信仰**	☐No Religion 沒有 ☐ Buddhism 佛教<br>☐ Catholic 天主教 ☐ Christian 基督教<br>☐ Hinduism 印度教 ☐ Muslim 回教<br>☐ Others 其他: (specify 請註明)________________
**6) Number of biological children 子女數目**	Number of sons 兒子數目: ________<br>Number of daughters 女兒數目: ________
**7) Living arrangement 居住安排**	☐ With family 和家人住 ☐ Alone 獨居<br>☐ Others 其他: ____________________(please specify 請註明)
**8) Monthly household income/ 家庭每月總收入**	☐ <10,000 HKD ☐20,000-29,999 ☐40,000-49,999<br>☐ 10,000-19,999 ☐30,000-39,999 ☐ ≥ 50,000
**9) Receiving aid from government?/有沒有使用政府的津貼?**	☐沒 No 沒有 ☐ Yes 有

<table>
<tr><td>10) Health insurance type 正在使用哪種類型的醫療保險?</td><td colspan="2">☐ None 沒有
◻ Public/Work-related insurance 公共/僱員保險
☐ Private insurance 私人保險</td></tr>
<tr><td>11) Your Age 年齡</td><td colspan="2">_________years 歲</td></tr>
<tr><td>12) Spouse/Partner Age 配偶年齡</td><td colspan="2">_________ years 歲 _______NA</td></tr>
<tr><td>13) Number of children wanted 想要的子女數目</td><td colspan="2">Number of sons 兒子數目: _________
Number of daughters 女兒數目: _________</td></tr>
<tr><td>14) Spouse had cancer/ 我的配偶曾經患有癌症</td><td colspan="2">◻ No 沒有 ◻ Yes 有 ◻ NA</td></tr>
<tr><td>15) I have had cancer/我曾經患有癌症</td><td>☐ No 沒有
(Go to Part 2) (請回答第二部分)</td><td>☐ Yes 有
If YES, please answer the following questions 如有，請回答以下問題</td></tr>
<tr><td></td><td></td><td>a) Year of first diagnosis 首次確診年份
____

(b) Type of cancer/ 癌症類型:
◻ Breast 乳癌 ◻ Lung 肺癌
◻ Colon 結腸癌
◻ Others 其他:
(specify 請註明)___________

(c) Stage at diagnosis/癌症確診時的期數
◻ Stage I ◻ Stage III
◻ Stage II ◻ Stage IV
◻ Not sure
◻ Others 其他:
(specify 請註明)___________

(d) Treatment received/ 治療方法
(can choose more than one answer)
(可選擇多個答案)
☐ Surgery 手術 ☐ Radiotherapy 放射治療
☐ Chemotherapy 化療
☐ Target therapy 標靶治療
☐ Others 其他: __________(specify 請註明)</td></tr>
</table>

16) Will you consider fertility preservation if your disease is known to have
如果你所需的治療將會出現以下情況,你會否考慮進行保存生育治療?

a. more than 1in 3 chance of causing infertility?<br>超過三分之一的機會導致不育	□No 不會	□Yes 會
b. about 1 in 5 chance of causing infertility?<br>五分之一的機會導致不育	□No 不會	□Yes 會
c. less than 1 in 20 chance of causing infertility?<br>導致不育的機會少於二十分之一	□No 不會	□Yes 會

17) What are the factors would you consider when deciding for fertility preservation? 你於決定進行保存生育治療時會考慮哪些因素? (Can choose more than one answer) (可選擇多個答案)

Your age 年齡	□No 不會	□Yes 會
Time available before start of cancer treatment<br>治療開始前所擁有的時間	□No 不會	□Yes 會
Type of cancer 癌症的類型	□No 不會	□Yes 會
Prognosis of cancer or medical condition 癌症的預後或疾病的狀況	□No 不會	□Yes 會
Financial resources available 可用資源	□No 不會	□Yes 會
Desire to have children 擁有孩子的渴望	□No 不會	□Yes 會
Marital status 婚姻狀況	□No 不會	□Yes 會
Cost 費用	□No 不會	□Yes 會
Religion 宗教	□No 不會	□Yes 會
Others 其他: ________________(please specify 請註明)	□No 不會	□Yes 會

Part 2: Awareness about fertility preservation
第二部分**:** 對於保存生育能力的認知

18) Which of the following fertility treatments have you ever heard of? (check all that apply):
閣下聽過哪種保存生育能力的治療方法? (請選擇所有適用)

a. Fertility-sparing surgeries 保存生育手術	☐存 No 沒有	☐ Yes 有	☐ Unsure 不知道
b. Radiation shielding 輻射屏蔽	☐射 No 沒有	☐ Yes 有	☐ Unsure 不知道
c. Sperm freezing 凍存精子	☐存 No 沒有	☐ Yes 有	☐ Unsure 不知道
d. Oocyte freezing 凍存卵子	☐存 No 沒有	☐ Yes 有	☐ Unsure 不知道
e. Embryo freezing 凍存胚胎	☐存 No 沒有	☐ Yes 有	☐ Unsure 不知道
f. Ovarian tissue or testicular tissue freezing 凍存卵巢織或睾丸組織	☐存 No 沒有	☐ Yes 有	☐ Unsure 不知道

19) How did you FIRST become aware of fertility preservation procedures? /閣下從何處得知保存生育能力的治療方法?

☐ From relatives/friends 從親戚/朋友得知
☐ From media/news 從媒體/新聞得知
☐ From doctors/nurses 從醫生/護士得知
☐ From news paper 從報紙得知
☐ From website 從網站得知
☐ From magazines 從雜誌得知
☐ Others 其他 (please specify 請註明) :______________

20) Have ***your friends/relatives*** undergone any of the following fertility preservation treatments shown above? / 閣下的朋友/親戚是否曾接受過以下任何一種保存生育的治療？
☐No 沒有 ☐ Yes 有 ☐ Unsure 不知道

Part 3: History of Medical Treatments and Counseling /第三部分**:** 治療和諮詢的經驗

21) Do you know that treatment for some diseases can have an adverse effect on fertility?
閣下知不知道有些疾病的治療可對生育有不利的影響嗎? ▫ No 不知道 ▫ Yes 知道

22) Do you want your doctor to discuss the options of fertility preservation to you if your treatment for your cancer disease has
如果閣下所需的治療將會有以下情況, 閣下是否希望你的醫生給你討論保存生育的治療方法?

a. more than 1in 3 chance of causing infertility?<br>超過三分之一的機會導致不育	No 不希望	Yes 希望
b. about 1 in 5 chance of causing infertility?<br>五分之一的機會導致不育	No 不希望	Yes 希望
c. less than 1 in 20 chance of causing infertility?<br>導致不育的機會少於二十分之一	No 不希望	Yes 希望

23) Before cancer treatments, were you counseled on ANY of the following fertility preservation methods (please check all that apply)? 在進行癌症治療之前，閣下是否曾接受過以下任何保存生育方法的諮詢？

- ☐ Sperm freezing 凍存精子
- ☐ Oocyte freezing 凍存卵子
- ☐ Embryo freezing 凍存肧胎
- ☐ Freezing ovarian/testicular tissue 凍 存卵巢織或睾丸組織
- ☐ Unsure if I received for any of these methods 不知道 / not available 不適用
- ☐ Fertility-sparing surgeries 保存生育手術
- ☐ Radiation shielding 輻射屏蔽

24) Have YOU ever UNDERGONE any of the following fertility preservation methods?
閣下是否曾接受過以下任何一種保存生育的治療？

a. Fertility-sparing surgeries 保存生育手術	☐存 No 沒有	☐有 Yes 有	☐ Unsure 不知道
b. Radiation shielding 輻射屏蔽	☐射 No 沒有	☐有 Yes 有	☐ Unsure 不知道
c. Sperm freezing 凍存精子	☐存 No 沒有	☐有 Yes 有	☐ Unsure 不知道
d. Oocyte freezing 凍存卵子	☐存 No 沒有	☐有 Yes 有	☐ Unsure 不知道
e. Embryo freezing 凍存肧胎	☐存 No 沒有	☐有 Yes 有	☐ Unsure 不知道
f. Freezing ovarian/testicular tissue 凍 存卵巢織或睾丸組織	☐卵 No 沒有	☐有 Yes 有	☐ Unsure 不知道

25) What is the SINGLE MOST IMPORTANT factor do you think you will consider when deciding for fertility preservation? (Please choose ONE only)
閣下認為哪一個因素是進行保存生育治療時最重要的單一因素? (請選擇 一個)

- ☐ Your age 年齡
- ☐ Religion 宗教
- ☐ Desire to have children 擁有孩子的渴望
- ☐ Marital status 婚姻狀況
- ☐ Cost 費用
- ☐ Type of cancer 癌症的類型
- ☐ Financial resources available 可用的财政資源
- ☐ Time available before start of cancer treatment 治療開始前所擁有的時間
- ☐ Others 其他: ______________(please specify 請註明)

26) My family would think that fertility preservation is a good idea<br>我的家人認為保存生育能力是一個好主意	☐ No 否	☐ Yes 是	☐ Unsure 不確定
27) It is important in my culture to have children<br>生兒育女在我的文化中佔很重要的位置	☐ No 否	☐ Yes 是	☐ Unsure 不確定
28) I plan to get more information about ART<br>我計劃獲取更多有關輔助生殖技術的信息	☐ No 否	☐ Yes 是	☐ Unsure 不確定
29) I plan to have fertility preservation services<br>我計劃進行保存生育治療	☐ No 否	☐ Yes 是	☐ Unsure 不確定

30) Would any of the following stop you from seeking fertility preservation services?
以下任何一項會阻止閣下尋求保存生育能力的服務嗎

a. Lack of money/lack of financial support<br>缺乏資金/缺乏財務支持	☐ No 不會	☐ Yes 會	☐ Unsure 不確定
b. Don't want to have additional surgery to preserve fertility 不希望因須保存生育能力而接受額外手術	☐ No 不會	☐ Yes 會	☐ Unsure 不確定
c. Might delay the start of cancer treatment<br>可能會延遲開始癌症治療	☐ No 不會	☐ Yes 會	☐ Unsure 不確定
d. Worried that hormones/drugs used in fertility preservation might make the cancer grow faster<br>擔心用於保存生育治療的激素/藥物可能會使癌症惡化	☐ No 不會	☐ Yes 會	☐ Unsure 不確定
e. Think that conceiving child is a bad idea after having cancer. 認為在患上癌症後, 生兒育女并不理想	☐ No 不會	☐ Yes 會	☐ Unsure 不確定

Part 4: Knowledge & Views on Fertility Treatment Policies
第四部分：關於生育治療政策的知識和觀點

31) Do you think the government or medical establishment should set an upper age limit on fertility preservation? 閣下是否認為政府或醫療機構應為保存生育治療設定年齡上限？:

☐下 Should be no upper age limit 沒有上限 ☐有 Up to age 40 上限為 40 歲

☐Up to age 50 上限為 50 歲 ☐ Up to age 35 上限為 35 歲

☐ Up to age 45 上限為 45 歲 ☐ Unsure 不知道

32) Do you think the government or medical establishment should set a LOWER age limit on fertility preservation? 閣下是否認為政府或醫療機構應為保存生育治療設定年齡下限？

☐Should be no lower age limit 沒有下限 ☐有 above 25 25 歲以上

☐ after puberty 青春期後 ☐ above 30 30 歲以上

☐ above 18 18 歲以上 ☐ Unsure 不知道

33) . Any adult can access fertility preservation treatment in public hospitals 任何成年人都可以在公立醫院接受保存生育治療	☐No 否	☐Yes 是	☐ Unsure 不知道
34) Any adult can access fertility preservation treatments in private clinics and private hospitals 任何成年人都可以在私人診所和私人醫院中接受保存生育治療	☐No 否	☐Yes 是	☐ Unsure 不知道
35) Paid gestational surrogacy is permitted in Hong Kong /香港准許商業代孕	☐No 否	☐Yes 是	☐ Unsure 不知道
36) Do you feel confident that you can find the necessary information about ART? /閣下是否有信心找到有關輔助生殖技術的必要信息?	☐No 沒有	☐Yes 有	☐ Unsure 不知道
37) Do you think that you can access fertility preservation services in Hong Kong? 閣下認為您可以在香港使用保存生育服務嗎？	☐No 不可以	☐Yes 可以	☐ Unsure 不知道

38) Do you agree that fertility preservation should be provided for the following reason?
閣下認為保存生育治療應為以下這些原因提供嗎?

a. Delayed family planning due to treatment of cancer 因癌症治療而延遲生育計劃	☐No 不應該	☐Yes 應該
b. Delayed family planning due to career development in females 女仕因事業發展而延遲生育計劃	☐No 不應該	☐Yes 應該
c. Delayed family planning due to career development in males 男仕因事業發展而延遲生育計劃	☐No 不應該	☐Yes 應該
d. Single women who wants to freeze their eggs when they are young before they find their other half 單身女性想在自己還沒找到另一半之前趁年輕時凍存卵子	☐No 不應該	☐Yes 應該
e. Single men who wants to freeze their sperm when they are young before they find their other half 單身男仕想在自己還沒找到另一半之前趁年輕時凍存精子	☐No 不應該	☐Yes 應該
f. Individuals who may have exposure to occupational hazards like radiation/ chemical exposure 可能接觸輻射或化學品的職業人仕	☐No 不應該	☐Yes 應該
g. Men with poor semen quality wants to have sperm frozen for future use 精液質量差的男性差希望有冷凍精子以供將來使用	☐No 不應該	☐Yes 應該
h. Homosexual/lesbian couples 同性伴侶	☐No 不應該	☐Yes 應該
i. Unmarried adults who want to be single-parents 想成為單親父母的未婚成年人	☐No 不應該	☐Yes 應該
j. Couple who wants to have embryos frozen for future use when it is time for second child. 夫婦欲冷存胚胎為將來生第二個孩子	☐No 不應該	☐Yes 應該

39. Do you think setting up a dedicated clinic/centre for fertility preservation is necessary in Hong Kong? 你認為香港必須設立可提供有關保存生育治療的專科診所或中心嗎?	☐No 不須要	☐Yes 必須

40. Do you think fertility preservation should be provided as a public service? 你認為保存生育能力應否以公共服務模式提供?	☐No 不應該	☐Yes 應該
41. Do you think the government should provide funding to set up a clinic/centre for fertility preservation? 你認為政府應否為成立保存生育治療的專科診所或中心而提供資金?	☐No 不應該	☐Yes 應該

Part 5: Perceived Service Needs for Fertility Preservation:
第五部分： 對保存生育服務的需求

42). Do you feel that you have enough information to make informed decisions about fertility preservation at this time? /閣下是否認為現時有足夠的資訊作出有關保存生育治療的決定？

□No 否　　□Yes 是

43) Which of the following materials do you think is useful when counselling patients for fertility preservation to enhance patient information gathering and decision making? (can choose more than one)/ 閣下認為以下哪些資料於保存生育治療輔導時能幫助患者更深入了解並作決定? (可選擇多於 1 項)

☐ Education pamphlets 教育小冊子　☐ Website 網站

☐ Talks/lectures/ symposiums 講座/講座/座談會　☐ Videos 影片

☐ Others 其他: ________________(please specify 請註明)

☐ None of the above required/ Not useful 不需要

44) What is the maximum delay in cancer treatment you would consider in order to attempt fertility preservation, including referrals, consultations, and total treatment time?
閣下願意為保存生育能力 （包括轉介，諮詢和治療） 而延遲癌症治療的最長時限為？

□Less than 2 weeks 少於兩週　□ 2-3 weeks 二至三週
□1-2 months 一至兩個月　□ 3 months or more 三個月以上

Part 6: Willingness to Pay for Fertility Treatments / 第六部分：為生育治療付款的意願

45) What is a reasonable price for: 閣下認為以下項目的合理價格範圍為：

a. the initial consultation & fertility preservation assessment 你有關保保存生育治療的最初的諮詢和評估	□<br>< $500	□<br>$500-999	□<br>$1000-1999	□<br>$2000+
b. Preliminary check (e.g. blood taking for ovarian reserve testing, semen analysis) 有關保存生育能的初步檢查 (例如卵巢儲備功能檢測和精液分析)	□<br>< $500	□<br>$500-999	□<br>$1000-1999	□<br>$2000+
c. *the assisted reproductive technology* (e.g. intrauterine insemination, in-vitro fertilization) for fertility preservation 閣有關保存生育能力的輔助生殖技術(例如宮腔內人工授精,體外受精+/-胞漿內人工授精)	□<br><$10,000	□<br>$10,000-29,999	□<br>$30,000-49,999	□<br>$50,000 more or
d. *the storage of frozen eggs/sperm/gonadal tissue per year* for fertility preservation 保存生育能力而進行的卵子/精子/卵巢織或睾丸組織凍存	□<br>< $1000	□<br>$1000-2999	□<br>$3000-4999	□<br>$5000+

46) Which of the following should the Government pay for (Check all that apply) *(or partial payment)*

☐ initial consultation & fertility preservation assessment 保存生育治療的最初的諮詢和評估

☐Preliminary check (e.g. blood taking for ovarian reserve testing, semen analysis) 有關保存生育能的初步檢查 (例如卵巢儲備功能檢測和精液分析)

☐ assisted reproductive technology procedures (e.g. in-vitro fertilization)

☐ storage of frozen eggs/sperm/gonadal tissue

Thank you! 謝謝!
