# Supplementary material for: Knowledge and intentions to use fertility preservation among urban Chinese cancer patients: A study from Hong Kong
Source: PLoS One. 2024 Sep 11;19(9):e0307715. doi: 10.1371/journal.pone.0307715 (PMC11389933; doi:10.1371/journal.pone.0307715)
Supplement: S2 Appendix — (PDF) [file pone.0307715.s002.pdf]

香港中文大學
The Chinese University of Hong Kong

2021-10-27

Professor Jean Hee Kim (SPHPC)
The Jockey Club School of Public Health and Primary Care

**Survey and Behavioural Research Ethics**
**Reference No. SBRE-21-0228**

Dear Professor Jean Hee Kim (SPHPC),

I write to inform you that the Survey and Behavioural Research Ethics Committee has granted approval in principle for you to conduct the surveys or observation of human behaviour by non-clinical means as declared in the application for the following research:

Principal Investigator:
Professor Jean Hee Kim (SPHPC)

Affiliated Department/Unit:
The Jockey Club School of Public Health and Primary Care

Project Title:
Knowledge of assisted reproductive technologies among patients attending informational sessions

Name of Grant/Funding (Reference No.):
NA

Kindly be reminded that you should also obtain approval from other research ethics committees within the University (e.g., Clinical Research Ethics Committee, Animal Experimentation Ethics Committee) if any parts of your research do not fall under the scope of our Committee. Thank you for your attention.

Yours Sincerely,

Ms. Eva Yeung
Secretary of Survey and Behavioural Research Ethics Committee
